# Editorial: Increasing sustainability in livestock production systems through high-throughput phenotyping approaches

**DOI:** 10.3389/fgene.2024.1403133

**Published:** 2024-04-05

**Authors:** Amanda Marchi Maiorano, Michela Ablondi, Yongliang Qiao, Juan Pedro Steibel, Yeni Liliana Bernal Rubio

**Affiliations:** ^1^ Department of Animal Science, Federal University of Uberlandia, Uberlandia, Brazil; ^2^ Department of Veterinary Science, University of Parma, Parma, Italy; ^3^ Australian Institute for Machine Learning, University of Adelaide, Adelaide, SA, Australia; ^4^ Department of Animal Science, Iowa State University, Ames, IA, United States; ^5^ Zoetis, Kalamazoo, MI, United States

**Keywords:** animal breeding, genomics, efficiency, machine learning, phenomics, precision livestock farming

Key goals of sustainable animal production systems are reduction on environmental impact, improvement on social acceptability and consumer perception, and increase on economic profitability while sustaining local communities. These goals can be achieved by making efficient use of animal feed sources, implementation of breeding programs focused on selection for efficiency, resilience, greenhouse gas emissions and adaptability, guaranteeing food security through improved human and animal health, improving animal welfare, and ensuring economic and societal relevance of animal production systems to local communities. Achievement of the listed goals and objectives requires a) measuring and analyzing inputs and outputs of animal production systems, including animal-specific indicators, economically relevant traits and environmental variables, and b) making data-informed management and selection decisions, aligned with breeding and economic goals of the farm.

For the measurement and analysis of the different animal production variables, the emergence of novel tools for on-farm data recording, as in the case of Precision Livestock Farming (PLF), has revolutionized phenotype recording systems. Nowadays, novel technologies enable phenotyping large number of animals, allowing to define new traits or indicators and accessing real-time data to make more informed decisions on the sustainability of the production system. Also, PLF constitutes a novel approach to study traits that are difficult to measure or define, such as those related to animal fitness, health and welfare, fertility, feed efficiency, disease resistance, and adaptability. However, the use of data generated by PLF sensors and its integration with other available information at the animal level such as genomic data remains a challenge. In that sense, new methodologies and prediction algorithms have been investigated (Pérez-Enciso and Steibel, 2021; Wang et al., 2022; Chafai et al., 2023), using approaches such as machine learning.

Gebreyesus et al. used machine learning to investigate predictive ability of body weight from 3D image data in dairy cows. Specifically, the authors explored the use of over 80,000 records of contour features from 3D images and body weight measurements to evaluated predictive performance of body weight in a combined multi-breed dataset composed with Danish Holstein and Jersey cows. The authors split the data into training and test sets and used metrics to evaluate predictive performance including Pearson’s correlation coefficient (r), the root mean squared error (RMSE), and the mean absolute percentage error (MAPE). Catboost, AdaBoost, random forest were the supervised learning techniques that resulted in the highest prediction performances. The increase in data size seems to be important to improve prediction accuracy. The authors reported low prediction errors, showing that prediction of body weight using image data in dairy cattle is fast and promising under commercial farm conditions. It represented the high-throughput phenotyping of complex trait using non-invasive imaging techniques with potential application to livestock phenomics. We suggest delving into the details of this particular paper.

Besides innovations targeting large-scale phenotyping, a deep understanding of the genome biology of new and routinely measured traits is a path to sustainable livestock production. A common example of the potential implementation of genomic technology to improve sustainability is genomic selection for greenhouse gas emissions and feed efficiency in cattle. Nonetheless, the available methods to record individual emissions and feed intake data are expensive, limiting their broad application, which highlights the need for new cost-effective indicators or correlated measures. Other examples are integrating phenotypic, genotypic, and environmental information to select animals less affected by heat stress; and addressing the genetic and epigenetic mechanisms of adaptation to local and regional conditions to optimize breeding and management strategies. The relevance of adaptability relies on the premise that selecting more adapted animals represents a reduction in production cost and improvement of welfare in temperate and tropical climate conditions with outdoor systems.

Adriaens et al. studied resilience indicators and milk yield based sensor features for cows at different herds and from different breeds. What motivated the authors was the need for an agricultural transition towards more diverse and low-input dairy farming systems with animals best suited to the specific environmental conditions. The authors described that, in less intensive dairy systems, cows are kept outdoors and might be exposed to more environmental disturbances such as variable feed quality or weather extremes exposure. The authors also investigated highly specialized and intensive dairy farms.

The hypothesis put forward by Adriaens et al. suggests that certain breeds cope better with disturbances, and as a result, keep milk production more constant. On the other hand, the authors were not able to distinguished breed and herd effects because data structure limitations. For this reason, the situation mentioned before can also be interpreted as those animals being kept in environments with lower variability or disturbances. The results obtained by Adriaens et al. suggested that almost all breeds studied had fewer or less severe perturbations than Holstein cows, with highlights given to Jersey, Belgian Blue, and Meuse-Rhine-Yssel cows. Non-Holstein and crossed cows demonstrated more stable milk production with less severe perturbations. The conclusion drawn is that in the future, it might be possible to select within farm for features that best suit a farms’ breeding goal and environment in terms of milk yield resilience. Understanding how cows perform in their specific environment is fundamental to improve animal production and welfare. For a more comprehensive understanding of the study and its findings, we recommend to read the full article.

Van Goor et al. unraveled the genetic mechanisms behind piglet and fetal health during Porcine Reproductive and Respiratory Syndrome Virus (PRRSV) challenges going beyond our current scenario in swine production. Selection will be not just limited to observation and measurement but guided by the identification of genes related to traits, providing valuable results for the genetic improvement in pigs. The paper by Van Goor et al. addressed a topic of great concern to swine production, the PRRSV and its impact on thyroid hormone levels (T3 and T4) in piglets and fetuses. The authors pointed out to insights on the genomic control of phenotypes associated with host resilience and susceptibility. The study involved analyzing serum samples from piglets and fetuses that were challenged with the Porcine reproductive and respiratory syndrome virus. The authors find that thyroid hormone levels following PRRSV infection are lowly to moderately heritable and had genetic correlations with viral levels and weight-related traits. Heritability estimates for rare traits such as fetal viral level during PRRSV infection and fetal brain to liver weight ratio can be found in this paper. Serum thyroid hormone levels of swine during disease challenge follow a genetic architecture with candidate genomic regions that explain different proportion of variance and are in overlap, mostly, with immune-related, transporter-related, and transcriptional regulators.

The articles accepted for publication cover a wide range of themes involving sustainability and efficiency in livestock production. The paper by Mariani et al. invites us to rethink the efficiency of cheese-making processes for a more sustainable chain at the various stages of process. The authors developed formulas involving milk components such as fat, protein, and casein to disentangle their role on cheese yield traits. Fresh cheese yield, total solids and water retained in the fresh cheese, and 60-day ripened cheese were used as response variables in the analysis including milk components and udder health indicators. Protein fraction presented relevance to understand the mechanisms that determine cheese yield, however the authors emphasized that detailed protein profile is not still routinely used in milk payment schemes and selection indices. In view of what the authors have discussed, it is clear that individual protein profile is relevant however the available methods to record this trait are still expensive and time-consuming, limiting its broad application.

Durunna et al. found higher core body temperature MESORs (Midline Estimating Statistic of Rhythms) in the Fall-Winter regime compared to the Winter-Spring regime and determined the heritability of these temperature parameters, suggesting their potential as genetic improvement tools for beef production. Data was collected from Angus steers in feeding trials during western Canadian winters, which represent cold stress conditions that impact individual feed/growth efficiency performance. Correlations between production efficiency measures and core body temperature were estimated to investigate whether an animal’s core body temperature can predict the production efficiency profile in cold environments.

For this Research Topic, we have approved the publication of 6 articles written by 35 different authors. The collective effort among researchers, farmers, policymakers, and communities in general is essential for the successful implementation of innovative practices. We encourage all the community to browse and read the articles encompassed in this Research Topic and reflect on how technology and science can drive a more sustainable and efficient livestock production.
